# Alteration of human blood cell transcriptome in uremia

**DOI:** 10.1186/1755-8794-6-23

**Published:** 2013-06-28

**Authors:** Andreas Scherer, Oliver P Günther, Robert F Balshaw, Zsuzsanna Hollander, Janet Wilson-McManus, Raymond Ng, W Robert McMaster, Bruce M McManus, Paul A Keown

**Affiliations:** 1 PROOF Centre of Excellence, Vancouver, BC, Canada; 2 James Hogg iCAPTURE Centre, Vancouver, BC, Canada; 3 Infection & Immunity Research Centre, Vancouver, BC, Canada; 4 Immunology Laboratory, Vancouver, BC, Canada; 5 Departments of Statistics, Vancouver, BC, Canada; 6 Medicine, Vancouver, BC, Canada; 7 Computer Science, Vancouver, BC, Canada; 8 Medical Genetics, Vancouver, BC, Canada; 9 Pathology and Laboratory Medicine, University of British Columbia, Vancouver, BC, Canada; 10 Spheromics, Kontiolahti, Finland; 11 Departments of Medicine, Pathology and Laboratory Medicine, University of British Columbia, Immunology Room 1559, Vancouver General Hospital, 855 W 12th Ave, Vancouver, BC V5Z 1M9, USA

**Keywords:** Gene expression profiling, Uremia, Chronic renal failure

## Abstract

**Background:**

End-stage renal failure is associated with profound changes in physiology and health, but the molecular causation of these pleomorphic effects termed “uremia” is poorly understood. The genomic changes of uremia were explored in a whole genome microarray case-control comparison of 95 subjects with end-stage renal failure (n = 75) or healthy controls (n = 20).

**Methods:**

RNA was separated from blood drawn in PAXgene tubes and gene expression analyzed using Affymetrix Human Genome U133 Plus 2.0 arrays. Quality control and normalization was performed, and statistical significance determined with multiple test corrections (qFDR). Biological interpretation was aided by knowledge mining using NIH DAVID, MetaCore and PubGene

**Results:**

Over 9,000 genes were differentially expressed in uremic subjects compared to normal controls (fold change: -5.3 to +6.8), and more than 65% were lower in uremia. Changes appeared to be regulated through key gene networks involving cMYC, SP1, P53, AP1, NFkB, HNF4 alpha, HIF1A, c-Jun, STAT1, STAT3 and CREB1. Gene set enrichment analysis showed that mRNA processing and transport, protein transport, chaperone functions, the unfolded protein response and genes involved in tumor genesis were prominently lower in uremia, while insulin-like growth factor activity, neuroactive receptor interaction, the complement system, lipoprotein metabolism and lipid transport were higher in uremia. Pathways involving cytoskeletal remodeling, the clathrin-coated endosomal pathway, T-cell receptor signaling and CD28 pathways, and many immune and biological mechanisms were significantly down-regulated, while the ubiquitin pathway and certain others were up-regulated.

**Conclusions:**

End-stage renal failure is associated with profound changes in human gene expression which appears to be mediated through key transcription factors. Dialysis and primary kidney disease had minor effects on gene regulation, but uremia was the dominant influence in the changes observed. This data provides important insight into the changes in cellular biology and function, opportunities for biomarkers of disease progression and therapy, and potential targets for intervention in uremia.

## Background

Chronic kidney disease (CKD) is a debilitating disorder with profound medical and societal consequences, characterized by a marked reduction in health, quality of life, societal functioning, productivity and survival [1-4]. Pleomorphic manifestations of uremia appear as renal function declines, and include impaired cognition and execution of higher function tasks; disordered neuromuscular function with muscle weakness, seizures and sensorimotor neuropathy; altered endothelial function with accelerated vascular disease; hematological alterations with anemia, platelet dysfunction and bleeding; endocrine and metabolic disorders typified by insulin resistance, gonadal dysfunction, hyperparathyroidism, bone disease and soft-tissue calcification; and disorders of innate and adaptive immunology with features of both inflammation and immune deficiency [1,2].

The features of uremia have been attributed to disordered homeostasis caused by altered synthetic functions, reduced excretion of biological end-products, and disordered fluid balance associated with failure of renal function. Retention solutes found at higher levels in uremic subjects have been identified as uremic toxins based on their association with uremic symptoms in animals and humans with renal failure, the resolution of these symptoms when levels of these compounds are lowered, and the toxic effects when these substances are added to cells or tissues in vitro [5,6]. However, despite extensive investigation of the biology of uremia, and the application of recent advances in proteomics technology to investigate the causality of this syndrome [7], the molecular understanding of the precise disturbances in the uremic syndrome remains incomplete.

The development of high-throughput microarray technology, permitting simultaneous measurement of changes in expression of multiple genes within the human genome, provides the opportunity for novel insight into disease processes and molecular pathways of biological dysfunction [8,9]. Recent advances have improved the sensitivity, specificity and accuracy of histological diagnosis using this technology, and the field of functional genomics is consequently a focus of intense investigation in many disease states [10-12]. The current study therefore examines the differential patterns of gene expression in normal subjects and patients with renal failure and outlines some of the principal biological alterations observed in the uremic state.

## Results

### Subjects

Demographic and clinical details of the 95 subjects are shown in Table 1. Subjects with stage 5 renal failure were selected to comprise a spectrum of primary disorders and treatment strategies. They were predominantly male, Caucasian and with a mean age of 47 years; 23% were pre-dialysis, 46% were receiving hemodialysis and 30% were on peritoneal dialysis. The principal causes of renal disease were glomerulonephritis, polycystic kidney disease, diabetes, and other defined disorders including hypertension, interstitial nephritis and renovascular disease. No subjects were receiving immunosuppressive or cytotoxic drugs. Twenty normal disease-free controls who completed a health survey and were receiving no prescription medication served as a comparator group. They were predominantly male, Caucasian and had a mean age of 42 years. Serum creatinine (658 ± 287, 95% C.I. 569-746 umol/L vs normal: 60-115 umol/L), and urea (25 ± 52 mmol/L, 95% C.I. 8.9-41.1 mmol/L vs normal: 2.5-6.4 mmol/L) levels were markedly increased in uremic subjects, while peripheral white blood count (7.45 ± 2.35, 95% CI 7.79-9.37 × 10^9^/L vs. normal: 4.0-11.0 × 10^9^/L), neutrophil count (4.79 ± 1.8, 95% CI 4.97-6.03 x10^9^/L vs normal: 2.0-8.0 × 10^9^/L), and lymphocyte count (1.62 ± 0.67, 95% CI 1.41-1.83 × 10^9^/L vs normal: 1.2-3.5 × 10^9^/L) were within normal limits.

**Table 1:** Demographic and clinical characteristics of study subjects

**Subjects**	**Uremia**	**Uremia**	**Normal**
	**Discovery**	**Validation**	**Controls**
Number	63	12	20
Age (years)	47 ± 11	47 ± 13	42 ± 11
Male (%)	42 (67%)	7 (58%)	12 (60%)
Treatment status			
Pre-dialysis	15 (24%)	2 (17%)	N.A.
Hemodialysis	28 (44%)	7 (58%)	N.A.
Peritoneal dialysis	20 (32%)	3 (25%)	N.A.
Ethnicity (%)			
Caucasian	47 (75%)	10 (83%)	16 (80%)
Asian	11 (17%)	2 (17%)	3 (15%)
African-american	2 (2%)	0 (0%)	0 (0%)
Other	4 (6%)	0 (0%)	1 (5%)
Primary Disease (%)			
Glomerulonephritis	27 (43%)	5 (41%)	N.A
Diabetes	5 (8%)	2 (17%)	N.A
Polycystic Kidney Disease	11 (17%)	0 (0%)	N.A
Other	20 (31%)	5 (42%)	N.A

### Gene expression

**Figure 1:**
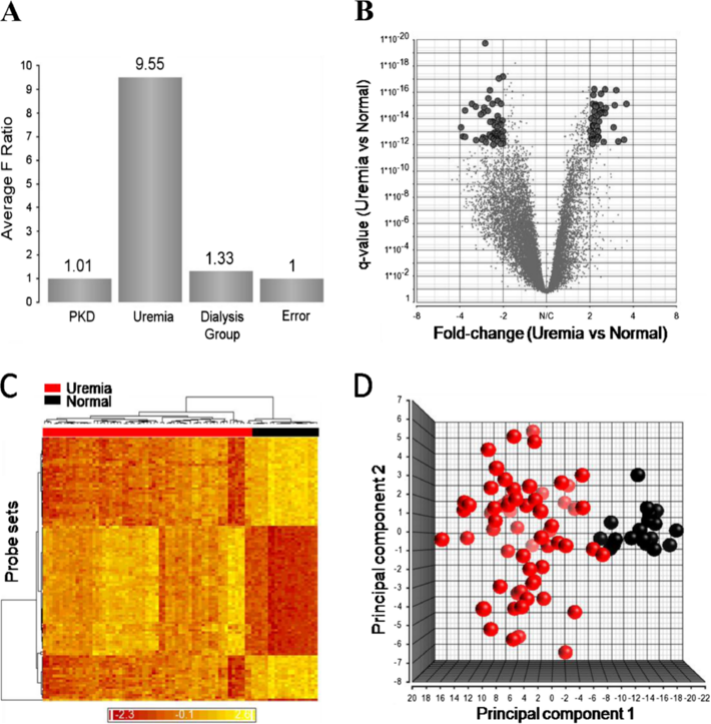
**Differential expression of probe sets between uremic and normal subjects detected by micro-array analysis.** (**A**) Sources of variation estimated in a multifactorial ANOVA model. The y-axis represents signal to noise ratio of the factors. (**B**) Volcano diagram showing magnitude and direction of change in gene expression. Grey points indicate the probe sets identified by ANOVA alone, and black points indicate the 110 probe sets with a qFDR < 1x10E-12 and |FC| > 2. (**C**) Unsupervised cluster analysis comparing uremic and normal subjects (squared Euclidean distance, average linkage). Each column represents an experimental subject while each row indicates a probe set. The color in each cell represents standardized log2-gene expression values, red being low and yellow high. (**D**) Principal component analysis showing separation of uremic and normal subjects.

Gene expression was profoundly altered in the uremic subjects. Approximately 25% (n = 12,933) of transcripts in the discovery cohort, reflecting 9,165 unique genes, were differentially expressed with a false discovery rate (qFDR) < 0.05 compared to normal controls. Fold change (FC) values ranged from -5.3 to +6.8, and the majority of transcripts (65%, n = 8,442) were lower in uremia. Over one thousand transcripts (n = 1,237) had an absolute fold change ≥ 2, of which almost 87% (1,080) were lower in uremia. To identify the most significantly differentially expressed genes we selected probe sets with a qFDR < 1x10^-12^, and a fold change > 2. The magnitude and direction of differential expression of the 98 genes returned in the discovery cohort are shown in the volcano diagram in Figure 1b. Segregation of the uremic and normal subjects by hierarchical cluster analysis is shown in the heat map in Figure 1c, and in the principal component analysis in Figure 1d. A listing of the functionally annotated genes which are most highly altered is provided in Table 2.

**Table 2:** Most highly differentially expressed functionally defined genes in uremic subjects by comparison with normal controls

		**Discovery cohort**		**Validation cohort**	
**Symbol**	**Gene Title**	**qFDR**	**Fold change uremia vs normal**	**qFDR**	**Fold change uremia vs normal**
**Under-repesented**					
ATP2A3	ATPase, Ca++ transporting, ubiquitous	1.90E-20	-2.67	1.73E-13	-3.32
MESDC1	mesoderm development candidate 1	6.41E-18	-2.01	3.66E-11	-2.03
FBRSL1	fibrosin-like 1	8.79E-18	-2.15	4.34E-14	-2.63
RNF19B	ring finger protein 19B	6.82E-17	-2.48	2.35E-14	-3.52
ATPIF1	ATPase inhibitory factor 1	2.92E-16	-2.54	5.36E-09	-2.39
FKBP1A	FK506 binding protein 1A, 12kDa	4.40E-16	-2.19	4.45E-11	-2.58
ILF3	interleukin enhancer binding factor 3, 90kDa	7.46E-16	-2.07	2.46E-10	-2.84
RBBP4	retinoblastoma binding protein 4	7.63E-16	-3.30	2.53E-11	-4.31
PEBP1	phosphatidylethanolamine binding protein 1	7.63E-16	-2.38	4.01E-09	-2.40
CTBP1	C-terminal binding protein 1	1.26E-15	-3.11	9.65E-11	-3.92
HINT1	histidine triad nucleotide binding protein 1	1.46E-15	-2.71	1.05E-13	-3.17
KLHL24	kelch-like 24 (Drosophila)	2.41E-15	-3.69	5.34E-12	-4.60
ILF3	interleukin enhancer binding factor 3, 90kDa	2.58E-15	-2.56	6.41E-11	-2.92
KDM1B	lysine (K)-specific demethylase 1B	3.66E-15	-2.76	8.63E-09	-3.06
MTA1	metastasis associated 1	4.91E-15	-2.87	4.62E-14	-3.80
KCTD5	potassium channel tetramerisation domain containing 5	6.35E-15	-2.19	2.57E-09	-2.36
CCDC115	coiled-coil domain containing 115	1.23E-14	-2.15	5.87E-09	-2.51
SLC23A2	solute carrier family 23 (nucleobase transporters), member 2	1.42E-14	-2.02	2.02E-08	-1.94
ACAD8	acyl-CoA dehydrogenase family, member 8	1.67E-14	-2.37	1.83E-09	-2.80
RAB11FIP4	RAB11 family interacting protein 4 (class II)	1.76E-14	-2.40	3.87E-16	-4.25
RNF19B	ring finger protein 19B	1.80E-14	-2.47	7.16E-12	-3.17
NONO	non-POU domain containing, octamer-binding	2.60E-14	-2.09	3.78E-13	-2.64
TNRC6A	trinucleotide repeat containing 6A	3.10E-14	-2.07	1.04E-14	-2.94
NDUFB8	NADH dehydrogenase (ubiquinone) 1 beta subcomplex, 8, 19kDa	3.33E-14	-2.29	8.30E-14	-2.77
OGT	O-linked N-acetylglucosamine (GlcNAc) transferase (UDP-N-acetylglucosamine:polyp	4.75E-14	-3.93	2.10E-11	-4.57
ATP5C1	ATP synthase, H + transporting, mitochondrial F1 complex, gamma polypeptide 1	4.50E-14	-2.20	1.53E-09	-2.33
MARCH5	membrane-associated ring finger (C3HC4) 5	9.53E-14	-2.01	3.16E-07	-2.11
PPP1R8	protein phosphatase 1, regulatory (inhibitor) subunit 8	1.02E-13	-2.21	4.88E-08	-2.23
RALGAPB	Ral GTPase activating protein, beta subunit (non-catalytic)	1.21E-13	-2.30	2.45E-09	-2.08
IRF2	interferon regulatory factor 2	1.49E-13	-2.20	1.94E-08	-2.47
ESYT2	extended synaptotagmin-like protein 2	1.54E-13	-2.38	4.12E-09	-2.71
BHLHE40	basic helix-loop-helix family, member e40	1.64E-13	-2.49	5.93E-09	-3.17
RABGAP1	RAB GTPase activating protein 1	1.69E-13	-2.06	3.77E-09	-2.25
GABPB2	GA binding protein transcription factor, beta subunit 2	2.09E-13	-2.15	6.64E-10	-2.31
QKI	quaking homolog, KH domain RNA binding (mouse)	2.27E-13	-2.39	2.00E-08	-2.39
FLI1	Friend leukemia virus integration 1	2.37E-13	-3.81	1.29E-11	-7.39
RAB7A	RAB7A, member RAS oncogene family	2.46E-13	-2.24	1.40E-09	-2.78
PDCD4	programmed cell death 4 (neoplastic transformation inhibitor)	2.52E-13	-3.69	1.01E-10	-4.69
BCL9L	B-cell CLL/lymphoma 9-like	2.52E-13	-2.02	8.78E-10	-2.14
RNF166	ring finger protein 166	2.66E-13	-2.42	9.07E-10	-2.55
ACTL6A	actin-like 6A	2.77E-13	-2.76	1.04E-08	-2.90
S1PR1	sphingosine-1-phosphate receptor 1	3.22E-13	-2.33	7.38E-10	-2.49
GLUD1	glutamate dehydrogenase 1	3.35E-13	-2.24	1.06E-09	-2.36
C7orf64	chromosome 7 open reading frame 64	3.35E-13	-2.68	3.61E-13	-4.25
CCDC88C	coiled-coil domain containing 88C	3.36E-13	-2.27	6.82E-09	-2.17
CSAD	cysteine sulfinic acid decarboxylase	3.73E-13	-2.33	1.27E-11	-2.94
ADSS	adenylosuccinate synthase	4.02E-13	-2.80	2.72E-11	-4.46
SRSF1	serine/arginine-rich splicing factor 1	4.48E-13	-3.09	6.01E-10	-3.51
LOC93622	hypothetical LOC93622	4.87E-13	-2.28	1.31E-07	-2.63
ACLY	ATP citrate lyase	5.37E-13	-2.01	4.00E-10	-2.69
PRF1	perforin 1 (pore forming protein)	5.40E-13	-2.18	1.79E-10	-3.28
MAPK9	mitogen-activated protein kinase 9	6.36E-13	-2.64	3.61E-07	-2.46
KLF7	Kruppel-like factor 7 (ubiquitous)	8.27E-13	-2.01	4.73E-09	-2.68
PIK3IP1	phosphoinositide-3-kinase interacting protein 1	8.63E-13	-2.08	1.98E-11	-2.38
TRIB2	tribbles homolog 2 (Drosophila)	9.60E-13	-2.35	8.64E-08	-2.67
**Over-represented**					
MORN1	MORN repeat containing 1	5.75E-17	2.15	2.23E-14	2.92
FGF18	fibroblast growth factor 18	5.92E-17	2.55	1.73E-11	2.76
C14orf45	chromosome 14 open reading frame 45	1.59E-16	2.11	7.17E-13	2.69
ZNF205	zinc finger protein 205	7.63E-16	2.09	1.44E-14	3.99
ADARB1	adenosine deaminase, RNA-specific, B1	8.38E-16	2.20	1.24E-12	2.87
GLTSCR2	glioma tumor suppressor candidate region gene 2	8.95E-16	2.37	2.72E-12	3.12
SAP30L	SAP30-like	9.54E-16	2.98	1.28E-11	3.85
ODF3B	outer dense fiber of sperm tails 3B	1.07E-15	2.22	3.72E-12	2.57
SRCRB4D	scavenger receptor cysteine rich domain containing, group B (4 domains)	1.14E-15	2.16	6.71E-11	2.38
TNPO2	transportin 2	1.26E-15	2.20	1.19E-11	2.68
MAPRE3	microtubule-associated protein, RP/EB family, member 3	2.58E-15	2.35	1.82E-11	2.81
LOC100507328 LOC100508591	hypothetical LOC100507328 /// hypothetical LOC100508591	2.94E-15	2.14	1.04E-14	2.76
DUX4	double homeobox 4 /// double homeobox 4 like 2 /// double homeobox 4 like 3 ///	3.61E-15	2.41	9.36E-15	2.98
RUNX3	runt-related transcription factor 3	3.61E-15	2.49	1.30E-12	2.44
PCGF5	Polycomb group ring finger 5	3.66E-15	2.51	5.03E-15	3.96
GPR144	G protein-coupled receptor 144	3.82E-15	2.55	1.61E-13	3.01
MAPK8IP2	mitogen-activated protein kinase 8 interacting protein 2	5.30E-15	2.29	7.39E-13	2.72
ZFPL1	zinc finger protein-like 1	1.00E-14	2.05	1.34E-13	3.04
PLEKHG5	pleckstrin homology domain containing, family G (with RhoGef domain) member 5	1.52E-14	2.15	4.25E-11	2.45
ELAVL3	ELAV (embryonic lethal, abnormal vision, Drosophila)-like 3 (Hu antigen C)	1.65E-14	2.32	2.97E-11	2.48
FBXO44	F-box protein 44	3.33E-14	2.09	1.70E-14	3.02
UBE2J2	ubiquitin-conjugating enzyme E2, J2 (UBC6 homolog, yeast)	3.49E-14	2.13	9.72E-11	2.43
IL34	interleukin 34	3.52E-14	2.11	1.68E-10	2.37
DKFZp761P0212	hypothetical protein DKFZp761P0212	4.11E-14	2.24	6.49E-15	2.94
SLC25A37	solute carrier family 25, member 37	4.75E-14	2.93	5.93E-10	3.17
SCNN1A	sodium channel, nonvoltage-gated 1 alpha	8.43E-14	2.04	4.65E-13	2.60
SFTPC	surfactant protein C	8.69E-14	2.28	5.61E-12	2.55
PDLIM7	PDZ and LIM domain 7 (enigma)	1.64E-13	2.10	5.36E-12	2.33
ARMC5	armadillo repeat containing 5	2.56E-13	2.06	1.19E-14	2.67
PLEKHN1	pleckstrin homology domain containing, family N member 1	2.58E-13	2.30	2.08E-14	3.00
GNAS	GNAS complex locus	2.86E-13	2.27	1.67E-16	4.17
EPN1	epsin 1	2.87E-13	2.00	6.34E-14	2.58
BCL2	B-cell CLL/lymphoma 2	3.77E-13	2.07	1.67E-13	2.72
CHCHD5	Coiled-coil-helix-coiled-coil-helix domain containing 5	3.91E-13	2.08	8.24E-08	1.91
TFRC	Transferrin receptor (p90, CD71)	4.09E-13	2.13	4.14E-13	2.46
RPS11	Ribosomal protein S11	4.14E-13	3.46	4.53E-17	7.01
C17orf51	chromosome 17 open reading frame 51	4.14E-13	2.21	4.53E-17	4.13
LMNA	Lamin A/C	4.18E-13	2.23	2.78E-09	2.34
CYP11B2	cytochrome P450, family 11, subfamily B, polypeptide 2	4.98E-13	2.11	2.12E-13	3.02
TMEFF2	transmembrane protein with EGF-like and two follistatin-like domains 2	5.64E-13	3.15	1.84E-14	5.71
GP1BB	glycoprotein Ib (platelet), beta polypeptide	6.19E-13	2.49	1.52E-15	3.24
TRIM8	Tripartite motif-containing 8	8.35E-13	2.00	2.54E-10	2.36
VRTN	vertebrae development homolog (pig)	8.63E-13	2.08	2.22E-11	2.18

Genes were selected by conjoint filter to identify those with smallest qFDR and highest FC.

**Figure 2:**
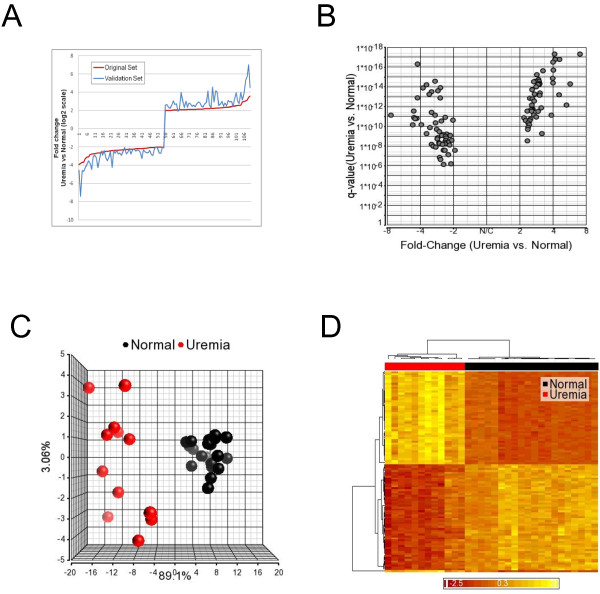
**Visualization of data in the Validation Cohort, showing differential expression, Volcano Plot, Principal Component Analysis and Hierarchical clustering of 100 most highly differentially expressed transcripts from the Discovery Cohort. A**: Fold change comparison. Fold changes were sorted by value in the discovery cohort (red line). The x-axis represents the numbered probe sets. Fold change direction is identical and in similar range for all probe sets in both cohorts. **B**: Volcano plot showing the qFDR and the fold changes for the 110 probe sets in the validation cohort after ANOVA. The qFDR and fold change are comparable in both cohorts. **C**: PCA utilizing the 110 probe sets from the validation cohort. The two groups are clearly separated indicating that the expression patterns of the transcripts are comparable in both cohorts. **D**: Hierarchical clustering of Normal and Uremic samples from the validation cohort based on 110 probe sets from the discovery cohort showing clear separation of both subject sets.

Analysis of the validation cohort confirmed these findings: 9,107 unique genes were differentially expressed with a qFDR < 0.05; FC values ranged from -15.6 to +9.7; and the majority of transcripts were again lower in uremia (71% overall, > 87% with |FC| ≥ 2). All 98 highly differentially expressed genes from the discovery cohort were again significantly altered in the same direction with a minimum fold change > 1.9 and a maximum qFDR of 3.6x10^-7^ (R^2^ = 0.960, p = 0.01) (Figure 2). The gene list, with qFDR values for both discovery and validation cohorts, is shown in Table 2.

Both dialysis and primary kidney disease (PKD) influenced gene expression in the study cohort, although this effect was small compared to the variation induced by the presence or absence of uremia. When the sources of variation in the dataset were estimated in a multifactorial ANOVA model, the presence or absence of uremia had the largest influence on the variation in the dataset (F ratio 9.55), while dialysis had a minor effect (F ratio 1.33) and the primary kidney disease, with polycystic kidney disease (PKD) as the reference group compared to the subgroups with renal disease secondary to diabetes mellitus (DM), glomerulonephritis (GN), and other etiologies (other) has the least influence (F ratio 1.01) (Figure 1a).

### Pathway analysis

**Figure 3:**
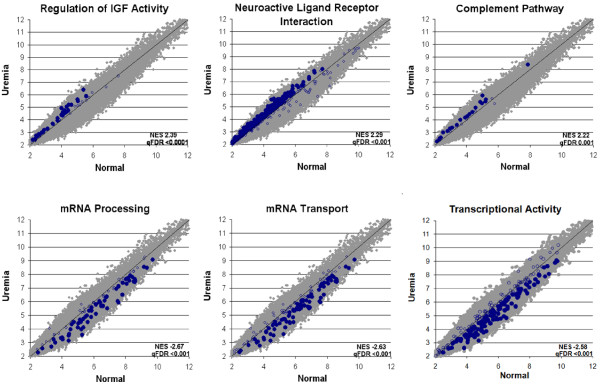
**Gene Set Enrichment Analysis (GSEA) by gene set permutation.** Blue dots represent enriched probe sets of the gene set, blue circles represent probe sets of the gene set that are not enriched, and grey dots represent all other probe sets on the array. X and Y axes are mean signal intensities in log2 scale. Source: http://www.broadinstitute.org/gsea/msigdb/index.jsp, MSigDB database v3.0 updated Sep 9, 2010.

The differentially expressed genes conformed to a broad array of biological pathways and gene networks that were under- or over-represented in uremia compared to normal subjects. Representative examples derived from gene-set enrichment analysis (GSEA) are shown in Figure 3. The functions most significantly decreased (q value < 0.01) involved mRNA processing, mRNA transport, and genes involved in transcriptional activity; others in this category included vesicle transport, transcription and RNA splicing, protein export and the unfolded protein response. The functions most significantly increased were Insulin-like Growth Factor (IGF) activity, neuroactive ligand receptor interaction, and the complement system; others included, the phospholipase C mediated cascade, serotonin receptors, and lipoprotein metabolism and lipid transport.

Highly altered genes (qFDR < 0.05; FC ≥ 1.25) showed important perturbations in key pathways of cellular function. The most profoundly dysregulated of these are shown in Table 3. Functions that were lower included the clathrin-coated vesicle endosomal pathway, the cytoskeletal remodeling pathway, RNA polymerase II transcription, the unfolded protein response, and protein export. The T-cell receptor signaling pathway, MHC-class II and the T-cell receptor alpha / beta heterodimer, the co-associated CD3 and CD4 molecules and a variety of downstream signaling components of the T-cell receptor pathway were importantly lower, as were those central to the immune synapse, the CD28 receptor pathway, the IL-2 response and signaling pathway. STAT3, SMAD3, MAPK1, c-Fos, Caspase -8 and -9, MICB, and WNT1 were markedly inhibited, influencing critical intracellular events of signal transduction, activation and regulation of cell proliferation. In contrast, MAP2K3, JAK1, amyloid beta 42, ubiquitin, and TNF beta were higher, influencing events involved in intracellular signaling, the inflammatory-related respiratory burst and the response to stress and injury. Expression of the erythropoietin receptor gene was elevated, although down-stream signaling steps through STAT1, 3 and 5 and others were repressed, while ligand receptor interaction encompassing events in hormone binding, ion channel activation, HDL-mediated lipid transport, histidine metabolism and phenylalanine metabolism were also higher.

**Table 3:** Principal gene pathways altered in uremia

**Principal gene pathways altered in uremia**	**p-value**	Ratio*	
Transport: Clathrin-coated vesicle cycle	8.039E-23	60 /	71
Cytoskeleton remodeling: TGF, WNT and cytoskeletal remodeling	2.990E-19	77 /	111
Cytoskeleton remodeling: Cytoskeleton remodeling	3.226E-17	70 /	102
Development: EPO-induced Jak-STAT pathway	2.658E-16	33 /	35
Translation: Regulation of EIF4F activity	2.083E-15	43 /	53
Chemotaxis: CXCR4 signaling pathway	2.445E-14	31 /	34
Development: GM-CSF signaling	4.953E-14	40 /	50
Immune response: T cell receptor signaling pathway	5.938E-14	41 /	52
Immune response: IL-2 activation and signaling pathway	1.410E-13	39 /	49
Oxidative phosphorylation	1.787E-13	66 /	105
Immune response : Immunological synapse formation	2.407E-13	44 /	59
Development: Flt3 signaling	2.595E-13	36 /	44
Signal transduction: Activation of PKC via G-Protein coupled receptor	5.244E-13	40 /	52
Cell cycle: Influence of Ras and Rho proteins on G1/S Transition	1.552E-12	40 /	53
Immune response: Role of DAP12 receptors in NK cells	4.346E-12	40 /	54
Immune response: BCR pathway	4.346E-12	40 /	54
Transcription: NF-kB signaling pathway	4.945E-12	32 /	39
Development: PIP3 signaling in cardiac myocytes	9.777E-12	36 /	47
Development: EGFR signaling pathway	1.026E-11	44 /	63

*# genes in list in pathway/# genes in pathway.

### Network analysis

**Figure 4:**
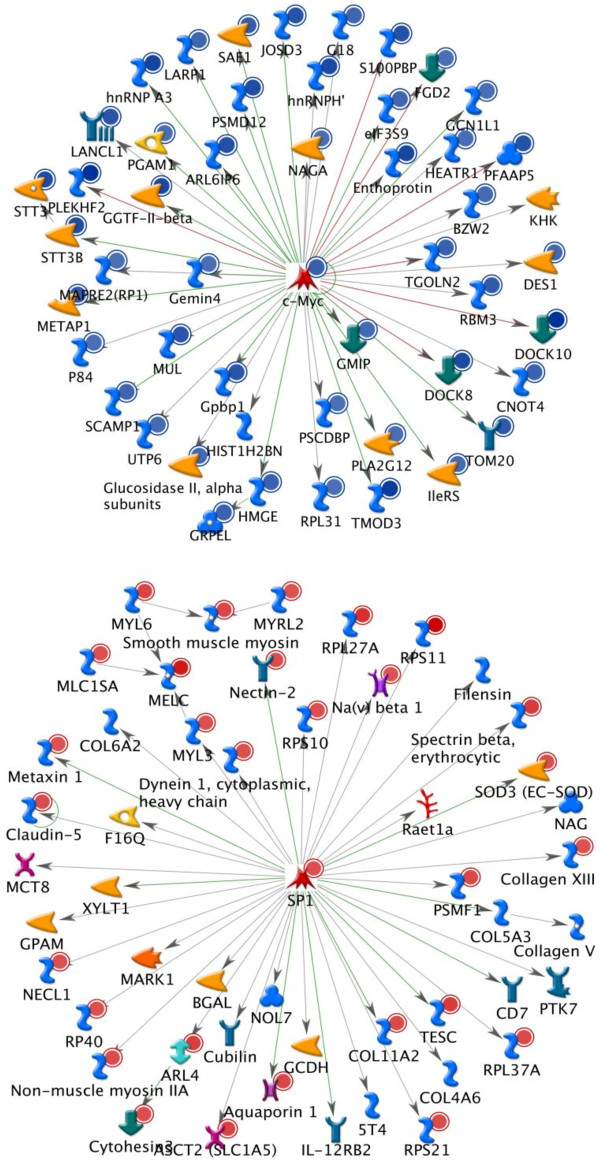
**Pathway analysis showing principal pathways altered in relation to the transcription factors c-Myc and SP1.** Blue wavy icons: generic binding proteins, yellow arrows: generic enzymes, green arrows: regulators. Blue dots: under-represented, Red dots: over-represented. The complete legend can be found at: http://ntp.niehs.nih.gov/ntp/ohat/diabetesobesity/Wkshp/MC_legend.pdf.

Differentially expressed genes in uremic subjects encoded a broad range of macromolecular functions and metabolic networks across all locations within the cell. Many of these diverse functions were regulated through key gene networks. Two representative networks demonstrating the central roles of cMYC (down-regulated) and SP1 (up-regulated) are shown in Figure 4. Other transcription factors playing central roles in regulating nuclear and cellular biosynthetic and metabolic processes included P53, AP1, NFkB, HNF4 alpha, HIF1A, c-Jun, STAT1, STAT3 and CREB1.

## Discussion

Chronic kidney disease is a global problem, with an estimated prevalence of more than 20% in those over 64 years of age [13] and health care cost approaching $2 billion per year in Canada and 7% of Medicare expenditures in the U.S. [14,15]. Dialysis may ameliorate the symptoms of uremia, but inadequate clearance of uremic toxins ultimately results in progressive illness manifest by chronic injury to the vascular tree, skeleton, neuronal networks and other critical bodily systems [1,2]. The European Uremic Toxin Work Group has listed more than 100 retained solutes that variably impair cellular function or survival and are important contributors in the expression of uremia [16]. Among these, small molecules that bind reversibly to serum proteins and “middle molecule” range proteins of 10-30KD are difficult to remove by conventional dialysis [5]. Many of the latter may become irreversibly altered through posttranslational modifications in the uremic environment, resulting in changes in structure and function [5].

The data reported here show that uremia is accompanied by profound changes in gene expression reflecting perturbation in many aspects of cell biology [17]. Genes encoding regulators of transcription, mRNA transport, protein synthesis, export and localization, and cell-cycle progression are lower, and transcripts associated with membrane lipid metabolism involving phosphotidylinositol 3,4,5; n-acyl sphingosine; ceramide and others are significantly lower in uremia. Cytoskeletal remodeling is markedly impaired, and expression of genes for the binding proteins talin and actin, critical structural components of intracellular microfilaments, regulators such as tuberin, and RAS-superfamily GTP-ases integral to cytoskeletal re-organization are substantially reduced. Interestingly, transcripts central to apoptosis pathways including the Fas receptor, FADD, Granzyme B and members of the caspases family are also reduced arguing against a principal role in premature cell death [18,19].

Among the complex endocrine changes associated with uremia [20], we observe that parathyroid hormone gene (PTH) expression is enhanced, consistent with the elevated hormone levels observed [1]. The Wnt signaling pathway is activated in hyperarathyoidism [21] and is strongly represented in the current dataset by probe sets including Casein kinase 1, Rac1, c-Fos, and p130. Smad2 and Smad4, TGFBR2 and other members of the TGF-beta and BMP pathways, among the most highly dysregulated probe sets in uremia, may reflect altered bone metabolism [22]. Expression of genes coding for the pituitary hormones was unchanged, while the prolactin releasing hormone (PRLH) gene was increased and prolactin regulatory element binding (PREB) gene reduced. Erythropoietin production is normally decreased in uremia. Possibly as a compensation to this, the erythropoietin receptor gene expression was significantly higher, while the down-stream signaling steps were repressed, perhaps contributing to the anemia of renal failure [1]. The effect of uremia on platelet function may be reflected by changes in the probe sets coding for PKCeta, Rac1, ATP2A3, and GP-IB (platelet glycoprotein I beta) and other members of the “platelet aggregation” network.

Insulin resistance is an important endocrine effect of uremia, and is believed to contribute to accelerated vascular disease and muscle wasting [23]. Although insulin binds normally to its receptor in uremia, and receptor density is unchanged, the transfer of insulin resistance by uremic serum suggests a direct contribution of uremic toxins. The data reported here indicates that insulin receptor gene (INSR) expression is modestly increased but the transcriptional level of insulin receptor substrate 2 (IRS2) is lower than normal. This cytoplasmic signaling molecule mediates the effects of insulin, acting as a molecular adaptor between diverse receptor tyrosine kinases and downstream effectors, and mice lacking IRS2 have a diabetic phenotype. Failure of post-receptor signaling has been noted as a fundamental mechanism of insulin resistance in uremic animals and in other disorders including injury, infection, aging and obesity and may reflect an important biological mechanisms in uremia [24].

Protein-calorie malnutrition is an important predictor of patient survival in uremia. Although the precise cause remains unclear, insulin resistance, inflammation, and elevated circulating levels of ghrelin and leptin have been implicated in this process [25-27]. While transcription of Ghrelin or Leptin genes was not altered, expression of both the leptin receptor overlapping transcript (LEPROT) and transcript-like 1 (LEPROTL1) was increased, which may influence leptin and GH receptor expression and their receptor-mediated signaling [28]. Growth factor and insulin-like growth factor (IGF) gene expression were unchanged, while IGF receptor-1 expression was suppressed and post-receptor signaling through the 14-3-3 protein complex was lower, which may influence protein synthesis, muscle and bone metabolism [29]. AKTIP was lower in uremia, consistent with the proposals that insulin resistance may promote muscle wasting by inhibition of PI3K/Akt leading to activation of caspase 3 and the ubiquitin-proteasome proteolytic [27]. Activation of the ubiquitin-proteosome system (UPS), caused by inflammation, acidosis and other factors is a feature of muscle wasting conditions including sepsis and uremia [30]. However, probe sets of the protein-degradation machinery, e.g. UBE2E1, USP32, UBE2Q2, and UBR3 were inhibited in uremia, indicating that evaluation of the ubiquitin-proteosome machinery requires more detailed investigation.

Uremia is characterized by a complex alteration in the immune response [31]. Systemic inflammation, manifest by elevations in inflammatory markers including C-reactive protein, interleukin-6, and tumor necrosis factor α [31], is accompanied by polymorph and monocyte dysfunction [32], and impaired cellular immunity with altered T cell function and proliferation [33]. The data here reflect many of these events at the genomic level. Gene expression associated with the complement pathway and oxidative metabolism is higher in uremia, while transcripts associated with the clathrin-coated vesicle endosomal pathway are markedly reduced consistent with a defect in phagocytosis. Key genes in the immune synapse and the T-cell receptor signaling pathway were reduced, including MHC-class II and the T-cell receptor alpha / beta heterodimer, the co-associated CD3 and CD4 molecules and a variety of downstream signaling components of the T-cell receptor pathway, the CD28 receptor pathway and the IL-2 response and signaling pathway.

Peripheral blood is a common matrix for investigation of human biology and biomarkers, but is subject to certain limitations which may influence the results observed. Fluctuation in peripheral formed elements may influence gene expression patterns, and while we have attempted to minimize this by selecting candidates whose peripheral blood counts resemble as closely as possible those of the normal control population this does not eliminate all bias. In addition, the presence of globin mRNA which represents up to 70% of the total expressed transcripts in peripheral blood, reduces the sensitivity of microarray analysis, particularly in detecting differences among genes transcribed at low levels [34-36]. Strategies to reduce globin mRNA were not employed in these studies, since preliminary data indicated the profound magnitude of the changes in uremia, but it is possible that this step may enhance the sensitivity of these results and define further critical biological alterations in the uremic state [34].

## Conclusions

In summary, the data presented show that uremia is accompanied by a marked change in expression of genes involved in a broad range of physiological processes [1,6]. Many of these genes appear to be coordinately regulated through networks whose activity is suppressed or enhanced by individual transcription factors. Recent work suggests that epigenetic regulation may exert an important influence in these changes, and that histone hypermethylation may contribute to both the reduced expression and increased inflammatory mechanisms observed in this setting [37,38]. These observations provide an important insight into the biology of the uremic syndrome and a foundation for more detailed proteogenomic exploration of uremic toxicity. They provide a foundation for exploration of biomarkers for measurement of treatment efficacy, and offer a starting point for identification of new therapeutic targets regulating gene effects to mitigate the consequences of this syndrome and restore biological homeostasis.

## Methods

### Study design

The study was conducted at the University of British Columbia and approved by the human ethics research board. A case-control design was employed to compare gene expression in patients with chronic renal failure and healthy controls. Patients with stage 5 renal disease aged 18 to 75 years, who were clinically stable awaiting renal transplantation, were not receiving immunosuppressive medications, and provided written informed consent were enrolled into the study. Patients were treated according to Canadian Guidelines for Chronic Kidney Disease [39]. Dialysis was instituted at a calculated GFR of less than 15 ml/min/m2; peritoneal dialysis was normally performed by continuous ambulatory peritoneal dialysis (CAPD) or a cycler, and hemodialysis (HD) was normally performed 3 times per week for an average of 12 hours. Normal controls of comparable age and gender who were screened to ensure freedom from known illness and medical therapy served as comparators.

### Study samples

Early morning, fasting, whole blood samples (5 ml) were drawn into PAXgene^TM^ tubes (Qiagen Inc) before dialysis or anticoagulation, and stored at -80° until analysis. Total RNA was extracted from the cells using a PAXgene^TM^ Blood RNA Kit, and the integrity and concentration determined using the Agilent 2100 BioAnalyzer (Agilent Technologies, Palo Alto, CA). Gene expression was analyzed at the CAP/CLIA certified Genome Core at the Children’s Hospital, Los Angeles, CA using Affymetrix Human Genome U133 Plus 2.0 arrays (Affymetrix Inc). Strategies to reduce globin mRNA were not employed in this study, since preliminary data demonstrated a marked difference between expression patterns in uremic and normal subjects. Quality of the samples, hybridization, chips and scanning was reviewed using the BioConductor packages Affy version 1.16.0 and affyPLM version 1.14.0. Data import, normalization and statistical analysis were performed using the Partek Genomics Suite, version 6.5 (Partek, St Louis, MI). RMA background correction and quantile normalization were applied followed by log2-transformation. An unsupervised raw expression filter was applied with a threshold of signal intensity of 6 in a number of samples equal to 75% of the smallest sample group. RNA samples for qPCR were reverse transcribed using SuperScript III First-Strand Synthesis kit (Invitrogen). qPCR assays were performed using gene-specific primers and Taqman gene expression assays (Applie Bioscience) on the ABI 7900 HT. Expression levels were normalized against β-actin.

### Statistical analysis

Statistical significance was determined by ANOVA, followed by multiple test corrections (qFDR). Probe sets were ranked by fold change after application of a qFDR threshold. A qFDR value < 0.05 was considered significant. Gene-set enrichment analysis (GSEA) was performed using GSEA software (http://www.broad.mit.edu/gsea). The dataset was not collapsed to gene symbols, probe sets were ranked by signal to noise metric, and the number of gene-set permutations was 1000. Biological interpretation was aided by knowledge mining using NIH DAVID (http://david.abcc.ncifcrf.gov/), MetaCore (http://www.GeneGo.com) and PubGene (http://www.Pubgene.org). Gene Ontologies and Networks in GeneGo MetaCore were prioritized based on their statistical significance with respect to the size of the intersection of the http://dataset and the set of genes/proteins corresponding to the Gene Ontology category or network (http://www.portal.genego.com/help/p-value_calculations.pdf).

### Research support

Research supported by Genome Canada with supporting grants from Novartis Pharma, Basle and IBM Canada.

## Competing interests

None of the authors have any conflict of interest in the research reported in this article.

## Authors’ contributions

PK, BM, RM and RN were the principal investigators for this research program. They obtained the research funding, designed the study, supervised the research program, analyzed the data and drafted the manuscript. AS, OG, RB and ZH conducted the data review and analysis, modeling and interpretation, and participated in the development of the manuscript. J W-M coordinated the study, supervised the clinical and analytical management teams, and participated in the development of the manuscript. All authors read and approved the final manuscript.

## Authors’ information

Paul A Keown, Bruce M McManus, W Robert McMaster and Raymond Ng are co-principal investigators in this research.

## Acknowledgements

**Biomarkers in Transplantation Group:** We are grateful to the following collaborators who participated in the current study by their valuable scientific advice or clinical contribution to the selection and management of the study subjects who formed the basis of this report: Alice Mui^1,3,a^, Tim Triche^b^, Gabriela Cohen Freue^1,5^, David Landsberg^7^, R. Jean Shapiro^7^, John Gill^7^, Jagbir Gill^7^, Olwyn Johnston^7^, Scott J. Tebbutt^1,2,7^.

a: Surgery, Vancouver, BC, Canada

b: Department of Pathology and Laboratory Medicine, Los Angeles Children’s Hospital, and University of California, Los Angeles, USA.

**Scientficic Advisory Committee**: We are grateful to the members of our Scientific Advisory Committee for their critical oversight and scientific guidance in this project: Professor. Kathryn Wood, Oxford University, UK; Ruedi Aebersold, Institute of Molecular Systems Biology, University of Zürich, Switzerland; John Quackenbush, Dana-Farber Cancer Institute, Boston, USA; Leigh Anderson, Plasma Proteome Institute, Washington, DC; Eric Olson, University of Texas – Southwestern, Dallas TX, Maria Rosa Costanzo, Midwest Heart, Edward Heart Institute, Naperville IL; Gunther Engel, Novartis Pharmaceuticals, Basel, and George Schreiner, Raven Biotechnologies, San Francisco, USA.

## Pre-publication history

The pre-publication history for this paper can be accessed here:

http://www.biomedcentral.com/1755-8794/6/23/prepub

## Data deposition

The data discussed in this publication have been deposited in NCBI's Gene Expression Omnibus (Edgar et al., 2002) and are accessible through GEO Series accession number GSE37171 (http://www.ncbi.nlm.nih.gov/geo/query/acc.cgi?acc=GSE37171).
